# Plectin reinforces vascular integrity by mediating crosstalk between the vimentin and the actin networks

**DOI:** 10.1242/jcs.172056

**Published:** 2015-11-15

**Authors:** Selma Osmanagic-Myers, Stefanie Rus, Michael Wolfram, Daniela Brunner, Wolfgang H. Goldmann, Navid Bonakdar, Irmgard Fischer, Siegfried Reipert, Aurora Zuzuarregui, Gernot Walko, Gerhard Wiche

**Affiliations:** 1 Department of Biochemistry and Cell Biology, Max F. Perutz Laboratories, University of Vienna, 1030 Vienna, Austria; 2 Department of Physics, Friedrich-Alexander-University of Erlangen-Nuremberg, 91052 Erlangen, Germany

**Keywords:** Vimentin, Plectin, Endothelial cell contractility, Vascular permeability, Shear stress

## Abstract

Mutations in the cytoskeletal linker protein plectin result in multisystemic diseases affecting skin and muscle with indications of additional vascular system involvement. To study the mechanisms underlying vascular disorders, we established plectin-deficient endothelial cell and mouse models. We show that apart from perturbing the vimentin cytoskeleton of endothelial cells, plectin deficiency leads to severe distortions of adherens junctions (AJs), as well as tight junctions, accompanied by an upregulation of actin stress fibres and increased cellular contractility. Plectin-deficient endothelial cell layers were more leaky and showed reduced mechanical resilience in fluid-shear stress and mechanical stretch experiments. We suggest that the distorted AJs and upregulated actin stress fibres in plectin-deficient cells are rooted in perturbations of the vimentin cytoskeleton, as similar phenotypes could be mimicked in wild-type cells by disruption of vimentin filaments. *In vivo* studies in endothelium-restricted conditional plectin-knockout mice revealed significant distortions of AJs in stress-prone aortic arch regions and increased pulmonary vascular leakage. Our study opens a new perspective on cytoskeleton-controlled vascular permeability, where a plectin-organized vimentin scaffold keeps actomyosin contractility ‘in-check’ and maintains AJ homeostasis.

## Abstract (precis)

**Summary:** Plectin-arranged vimentin scaffolds keep actomyosin contractility ‘in-check’ and maintain cell–cell junction homeostasis, providing a new perspective on cytoskeleton-controlled vascular permeability.

## INTRODUCTION

Weakened endothelial junctions lead to increased permeability and compromised vascular integrity causing haemorrhage, brain and pulmonary oedema, acute respiratory distress syndrome and increased susceptibility to stress induced injury (Maniatis and Orfanos, 2008). The interaction of adherens junctions (AJs) with the cytoskeleton, in particular through the AJ protein vascular endothelial (VE)-cadherin (also known as CDH5), plays a pivotal role in this context (Dejana et al., 2009). Mechanistically, it has been shown that increased actin-driven contractility generates tension that pulls cells away from each other, creating intercellular gaps and increasing permeability (Dudek and Garcia, 2001). This condition can be evoked upon thrombin, histamine, aldosterone release and, mechanically, upon stretch (Dejana et al., 2009; Shasby et al., 2002). In addition to actin filaments, recently the vimentin filament network has also emerged as a player in regulating endothelial permeability. Endothelial integrity of vimentin-deficient mice has been reported to be compromised and on the molecular level it has been shown that an intact vimentin filament network is crucial in counteracting endothelial barrier disruption in response to hypoxia (Liu et al., 2014, 2010; Nieminen et al., 2006). Thus, cytoskeleton integrity based on the balanced interplay of its components is emerging as key determinant of endothelial barrier function.

Plectin, one of the major cytoskeletal linker proteins, is a giant (>500 kDa) protein that primarily binds to intermediate filaments, but interacts with actin filaments and microtubules as well (Castañón et al., 2013). It anchors intermediate filaments at distinct organelles and strategic cellular sites, such as focal adhesions in fibroblasts and related structures in other mesenchyme-derived cells (hemidesmosomes and desmosomes in epithelial cells, and Z-disks in muscle fibres and cardiomyocytes). Through this anchorage (combined with filament cross-linking), plectin organizes the intermediate filament network such that it forms a cage-like structure encapsulating the nucleus, as shown for fibroblast and keratinocyte cell cultures. In plectin-deficient cells, a similar intermediate filament network organization is missing (Wiche and Winter, 2011). We have recently shown that intermediate filament anchorage is a necessary prerequisite for intermediate filaments to act as the physical constraint that the actomyosin system requires for generating internal tension. In the absence of intermediate filament anchorage, such as in P0 cell systems, insufficient cytoskeletal tension drives compensatory mechanisms upregulating actin stress fibres in a similar manner to in cells lacking intermediate filaments altogether (Gregor et al., 2014). In endothelial cells, the anchorage of vimentin filaments to focal adhesions has been shown to increase the adhesive strength and size of these structures (Bhattacharya et al., 2009; Tsuruta and Jones, 2003). Furthermore, Uehara and colleagues have shown that, in rat spleen endothelial cells, plectin predominantly occupies cell regions in which actin stress fibres were intermingled with vimentin filaments, and they suggested that these transverse actin stress fibres were crucial for sinusoidal blood vessel permeability (Uehara and Uehara, 2010).

Very little is known, however, on the molecular level, about how plectin functions in endothelial cells and what consequences plectin deficiency has on a functional endothelium. Given that most mutations in the plectin gene result in severe forms of the skin-blistering disease epidermolysis bullosa simplex associated with muscular dystrophy (EBS-MD), research on plectinopathies has been focused primarily on skin and muscle tissue. There are indications, however, that besides skin and muscle, vascular tissue is affected as well. For instance, severely haemorrhagic skin blisters have been reported for an EBS-MD patient (Bauer et al., 2001), in contrast to the majority of EBS cases, including those caused by keratin mutations, where blisters are filled with clear fluid (Wright et al., 1993). In other cases, arteriovenous malformations, and respiratory distress, in some cases associated with sporadic bleedings (Babic et al., 2010), have been observed (Mellerio et al., 1997; Schröder et al., 2002). Moreover, analysis of newborn plectin-knockout mouse pups revealed severe bleedings in their blistered paw regions (Andrä et al., 1997). Although these observations hinted towards increased fragility of stress-challenged blood vessels in the absence of plectin, hardly any attention has hitherto been given to the vascular system, mainly because of difficulties in discerning vascular from the dominating skin and muscular phenotypes.

In this study, we assessed the impact of plectin on the vasculature by using a combinatorial *in vitro* and *in vivo* approach that included the analyses of wild-type versus plectin-deficient endothelial cell systems and conditional plectin-knockout mice. Our study reveals a crucial role of plectin in maintaining vascular integrity through reinforcement of AJs. We show that vimentin intermediate filament networks mechanically restrain the contractile actomyosin system of endothelial cells in a plectin-dependent manner, enabling tight barrier formation. Our data highlight a hitherto unrecognized role of cytolinker proteins in vascular barrier protection upon mechanical stress exposure.

## RESULTS

### Plectin-null mice show vascular defects

**Fig. 1.**
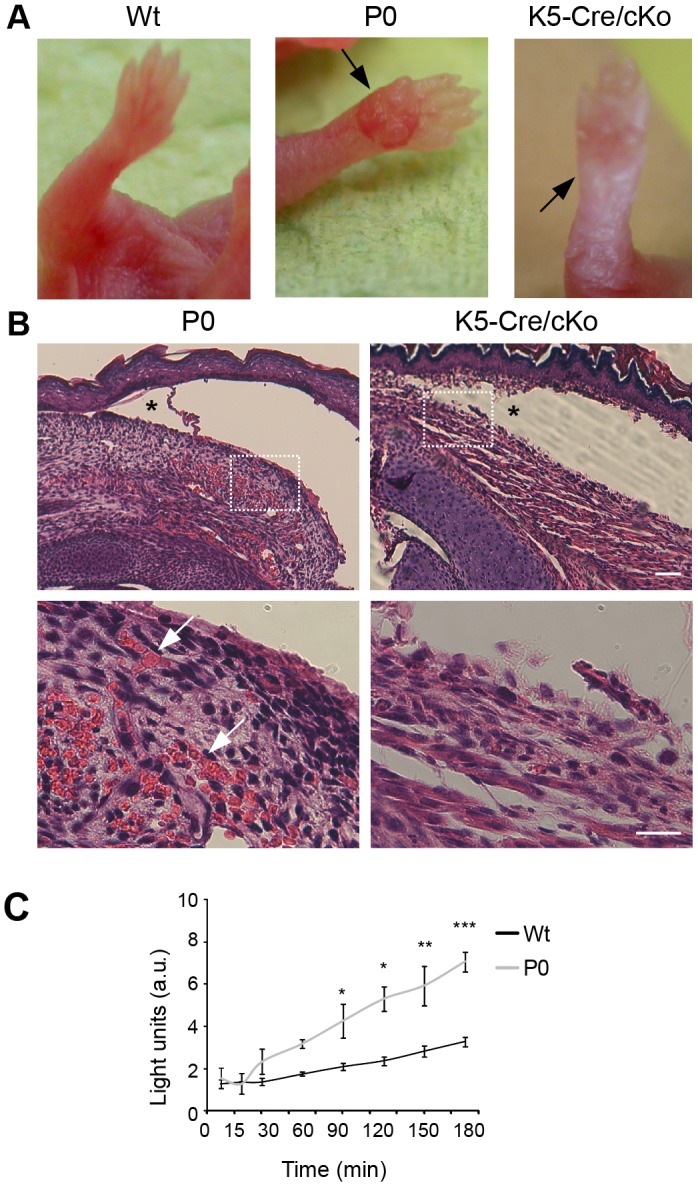
**Analysis of plectin-deficient mice and cell lines.** (A) Forepaws of newborn Wt mice, P0 mouse pups, and K5-Cre/cKO mice. Arrows point to a clear-fluid-filled blister and a haemorrhagic blister in P0 and K5-Cre/cKO mice, respectively. (B) Hematoxylin and Eosin (H&E) staining of P0 and K5-Cre/cKO skin in blister regions showing epidermal detachment at the level of the basal keratinocyte cell layer (asterisks). Boxed areas in the upper panels are shown as magnified images in the lower panels. Arrows, erythrocyte extravasations in dermal region of P0 mice (not observed in equivalent regions of K5-Cre/cKO mice). Scale bars: 50 µm (upper panel); 20 µm (lower panel). (C) Wt and plectin-deficient (P0) endothelial cells were grown on Matrigel-coated filters (3-µm pore size) in transwell inserts for 48 h. Transport of FITC–dextran (3 kDa) through the endothelial layers was measured by adding the substance to the lower (abluminal) side of the chamber and taking aliquots from the upper (luminal) part at the indicated time intervals. The fluorescence intensity of the aliquots was measured using a 96-well plate fluorimeter. Results are mean±s.e.m. from three independent experiments. a.u., arbitrary units. *P<0.05, **P<0.01, ***P<0.001 (Student's *t*-test).

To assess whether vascular defects contribute to haemorrhagic blister formation in plectin-deficient mice, we comparatively analysed the blistering phenotype of mice that were lacking plectin in all tissues (P0) and that of keratin 5-Cre conditional plectin-knockout mice (K5-Cre/cKO), where plectin deficiency is restricted to skin tissue (Ackerl et al., 2007). As depicted in Fig. 1A, both types of mice exhibited blister formation on their paws, however, only the ones of P0 mice were filled with blood, whereas the blisters of K5-Cre/cKO mice were filled with clear fluid (Fig. 1A, arrows). A histological examination showed that in both cases the blisters were forming between the dermis and the epidermis, typical of EBS. However, severe bleeding occurring in the dermis was revealed only in P0 mice (Fig. 1B, arrows); consequently, blister ruptures were accompanied by heavy bleeding only in this type of mutant mice. Of nine P0 animals stemming from nine different litters, all clearly showed blood-filled blisters, whereas the analysis of six K5-Cre/cKO mice from six different litters revealed five animals with blisters, all devoid of blood.

### Compromised barrier function of cell monolayers formed *ex vivo* by plectin-deficient endothelial cells

Haemorrhagic blister formation in P0 mice is suggestive of increased fragility and leakiness of the vasculature. To analyse this sort of phenotype on the cellular and molecular levels, we first aimed at isolating wild-type (Wt) and P0 endothelial cells. For this, primary endothelial cell cultures derived from Wt and P0 newborn mice were immortalized by polyoma middle T infection (Williams et al., 1988). In this way, two independent endothelial cell lines were established, one derived from kidneys (pT), the other from lungs (DH). As shown by immunoblotting of cell lysates, both lines expressed similar protein levels of the endothelium-specific marker protein VE-cadherin (Fig. S1). Furthermore, of the four major isoforms of plectin (P1, P1a, P1c and P1f) known to be expressed in a variety of different tissues (Castañón et al., 2013), two (P1a and P1) could positively be identified using isoform-specific antibodies (Fig. S2), whereas none of these isoforms could be detected in P0 cell lines (Fig. S2).

To assess whether plectin affects the permeability of endothelial cell monolayers forming the inner lining of blood vessels, we comparatively measured the permeation of FITC–dextran through cell monolayers formed by growing immortalized Wt and P0 endothelial (DH) cell lines to confluence on Matrigel-coated transwell filters. Whereas the dye only moderately penetrated Wt monolayers over time periods of 3 h, in P0 cells increased penetration was already evident after 1 h, reaching values twice as high as that of wild-type cells after 3 h (Fig. 1C). Similar results were obtained using primary cell cultures instead of cell lines (data not shown).

### Alterations of actin and vimentin network organization prompted by plectin deficiency lead to discontinuity of endothelial cell–cell junctions

**Fig. 2.**
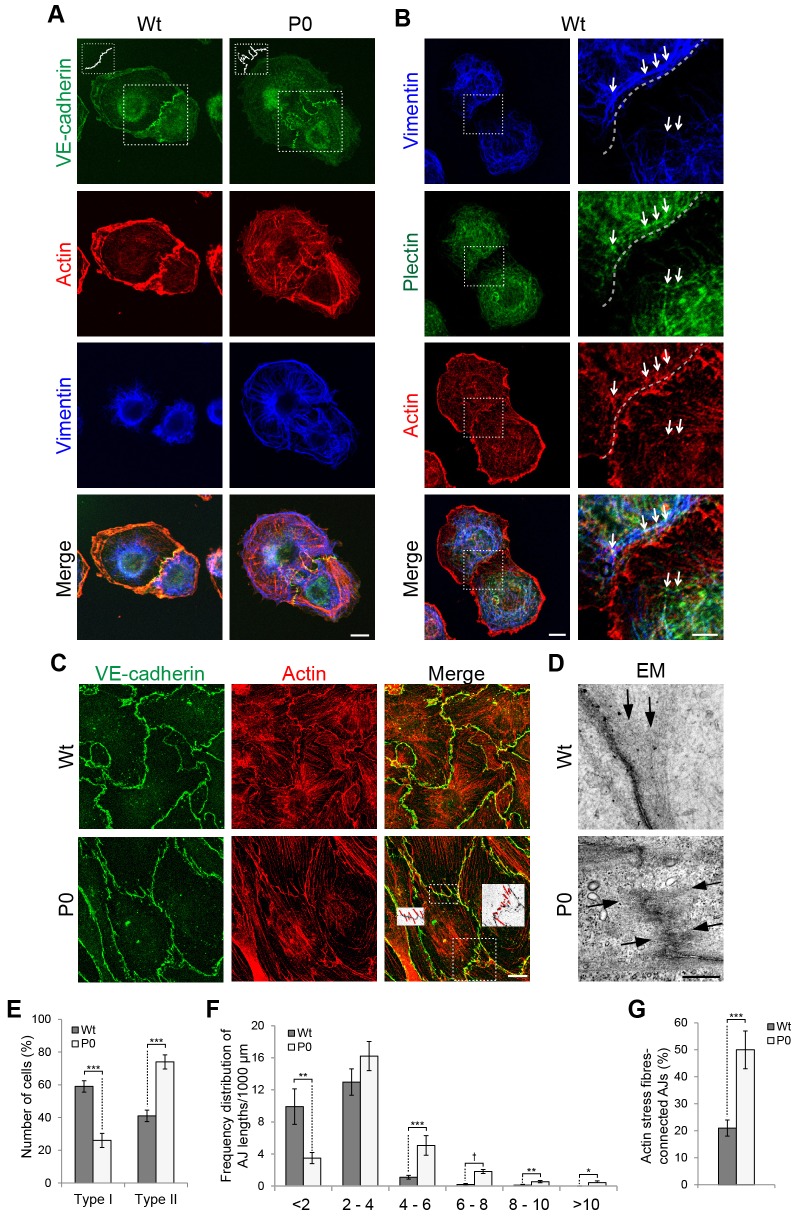
**Distortions of cell–cell junctional VE-cadherin in the absence of plectin.** (A) Triple immunolabelling of primary Wt and P0 cells using antibodies to VE-cadherin, actin and vimentin. To better visualize differences between Wt and mutant AJs, a free-hand reconstruction of VE-cadherin-marked cell–cell borders within the boxed areas is shown in insets in the upper left corner of the images in the top row. Scale bar: 10 µm. (B) Maximum intensity projection of confocal *z*-stacks after triple immunolabelling of primary Wt cells using antibodies to vimentin, plectin and actin (pseudocolours). Dashed boxes, cell–cell border regions shown enlarged in right panels. Dashed line, cell–cell border. Arrows, plectin patches aligning along vimentin filaments and partially colocalizing with actin patches. Scale bars: 10 µm (left column); 5 µm (right column). (C) Immunolabelling of confluent cell layers (∼24 h adhesion) formed from Wt and P0 endothelial cells using antibodies to VE-cadherin and actin. Areas with representative AJ distortions (spikes) in plectin-deficient cells are boxed (merged image). Black-and-white insets of the boxed regions show outlined (in red) VE-cadherin-positive spikes. Outlined structures were processed in ImageJ for length measurements; all marked structures were additionally scored for association with actin stress fibres (see G). Scale bar: 10 µm. (D) Transmission electron microscopy of endothelial cell–cell junctions in monolayer cultures of Wt and P0 endothelial cell lines. Arrows, actin stress fibres. Scale bar: 500 nm. (E) Percentage of primary cells (in randomly chosen optical fields) displaying pronounced cortical actin along AJs and sparse actin stress fibres (type I) versus cells lacking cortical actin along AJs but displaying pronounced actin stress fibres (type II); representative examples are shown in A. Results are mean±s.e.m. from five independent experiments, in total ∼350 cells. (F) Frequency distribution of AJ lengths per 1000 µm. Results are mean±s.e.m. (>900 junctions were measured per genotype from three independent experiments). (G) Junctions distinguished by actin stress fibres connections were counted in randomly chosen optical fields and their proportion of total junctions counted was determined for each genotype. Results are mean±s.e.m. (>90 junctions from three independent experiments). *P<0.05, **P<0.01, ***P<0.001, ^†^*P*<0.0001 (Student's *t*-test).

To gain insights into the mechanism how plectin affects the permeability of endothelial cell monolayers and consequently of blood vessels, we assessed the organization of cell–cell junctions along with that of actin filament and vimentin intermediate filament networks in Wt and P0 cells, using triple immunofluorescence microscopy. Monitoring the endothelium-specific cell–cell junctional marker protein VE-cadherin in cells at early stages of AJ formation (∼2 h of cell adhesion), we found dramatic differences between the two cell types. In contrast to the straight and in-line arrangement of AJs in Wt cells, AJs in P0 cells showed intermittent and staggered patterns, often resembling spikes extending into the cell interior (Fig. 2A). Deviations from the continuous and linear appearance of cell–cell junctions as extreme as those shown in the inset of Fig. 2A (top row, right panel) were observed in ∼25% of P0, but only ∼3% of Wt cell couples. In their appearance, AJs in P0 cells resembled a previously reported dynamic and more permeable pool of AJs (referred to as discontinuous AJs), the formation of which is promoted by actin stress fibres (Millan et al., 2010). In line with this, we observed a high abundance of actin stress fibres in P0 cells, contrasting the prominent cortical actin network and the rather delicate and sparse actin stress fibres of Wt cells (Fig. 2A, second row). Consistent with its role in stabilizing linear (continuous) AJs, cortical actin showed a high degree of colocalization with VE-cadherin in junctional areas of Wt cells (Fig. 2A, merged image). A statistical evaluation obtained by subdividing cell populations into two categories, one (type I) with cortical actin in cell–cell border areas, the other (type II) without cortical actin in junctional areas but with prominent actin stress fibres instead, revealed that the majority of Wt cells (∼60%) belonged to category type I, whereas ∼75% of P0 cells to category type II (Fig. 2E).

Monitoring vimentin networks in primary Wt cells, we observed the formation of vimentin cage-like structures encapsulating the nucleus (Fig. 2A, third row; and 2B, top row), similar to observations made in fibroblasts (Gregor et al., 2014; for reviews see Wiche and Winter, 2011; Wiche et al., 2015). In contrast, in P0 cells, vimentin filaments extended to the cell periphery, fully intruding the regions of cell–cell junctions (Fig. 2A). Thus, in Wt cells, the vimentin filament network terminated either distantly from the cell border, or filaments were found to run parallel and near to the border, whenever cell nuclei were situated close to the borders (Fig. 2B, top row; compare upper and lower cell shown in panel). In both cases, vimentin filaments were decorated by plectin, aligning in its typical ‘beads on a string’-like fashion along the filaments (Osmanagic-Myers et al., 2006) and also partially colocalizing with actin patches (Fig. 2B, arrows). Thus, we hypothesize (without excluding other possibilities) that, similar to the situation in other cell systems, plectin-mediated organization of vimentin filaments in a central cage-like structure surrounding the nucleus constrains the filaments from direct contacts with cell–cell border regions. In confluent cell layers (24 h adhesion), VE-cadherin-positive spikes and actin stress fibres were still more prominent in P0 compared to Wt cells, albeit the continuity of cell–cell junctions in general was higher under these conditions (Fig. 2C). Increased discontinuity of cell–cell junctions in P0 cells could also be observed after immunolabelling for the tight junction (TJ) protein ZO-1 (also known as TJP1), indicating TJs were affected by plectin-deficiency in a similar fashion (Fig. S3A); changes in ZO-1 protein levels were not observed (Fig. S3B). The highly irregular morphology of P0 cell–cell junctions could be resolved also on the ultrastructural level (Fig. 2D), where abundant perpendicularly orientated actin stress fibres seemingly ending at cell–cell junctions also were apparent (Fig. 2D, lower panel, arrows). In contrast, actin fibres in Wt cells mainly ran parallel to the predominantly linear cell–cell junctions (Fig. 2D, upper panel, arrows).

To statistically evaluate these phenotypic alterations, we measured the lengths of individual VE-cadherin- and ZO-1-positive spikes as outlined in the insets of Fig. 2C, assigned them to different length categories, and determined the frequency distribution of these categories per 1000 µm of analysed cell perimeters in different, randomly chosen optical fields. As expected, the numbers of elongated VE-cadherin- and ZO-1-positive spikes (>4 µm) were significantly increased in P0 cells (Fig. 2F; Fig. S3A) and so was the proportion of actin-stress-fibre-connected AJs (Fig. 2G). Reducing actin stress fibres by treatment of cells with the Rho-dependent kinase (ROCK) inhibitor Y-27632, led to smoother, more curved cell edges in both cell types, roughly abrogating the differences between Wt and mutant cells. As revealed by statistical evaluation there was a strong increase in shorter AJs (<4 µm, compare with Fig. 2F) observed for both cell types, whereas in the long (>6 µm) and short (<2 µm) length ranges, differences still persisted, possibly due to the more abundant residual actin stress fibres in P0 cells (Fig. S4A). When protein levels and localisation of the RhoA inhibitor GAP190 and of the activator GEF Syx (also known as PLEKHG5) (Ngok et al., 2012; Wildenberg et al., 2006) were analysed, no differences between Wt and P0 cells were found, implicating that plectin was not directly affecting these actin stress fibres-regulating signalling molecules (Fig. S4B,C).

**Fig. 3.**
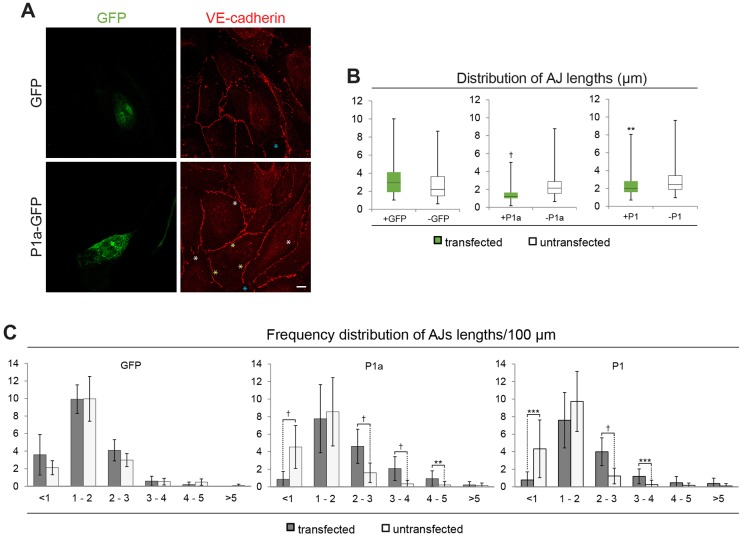
**Rescue of AJ distortions in P0 cells by forced expression of plectin isoforms.** Cell cultures were transfected with either empty vector (GFP), or vectors encoding GFP-tagged versions of P1a (P1a–GFP) or P1 (P1–GFP). (A) Representative images of P1a–GFP- and GFP-transfected cells immunolabelled with antibodies to VE-cadherin. Yellow and white asterisks, transfected–untransfected and untransfected–untransfected cell borders, respectively; blue asterisks, no cell–cell border. Scale bar: 10 µm. (B) Distribution of AJ lengths measured (ImageJ) in subconfluent (transfected and untransfected) P0 cells presented as a box plot diagram. The box represents the 25–75th percentiles, and the median is indicated. The whiskers show the range. >150 junctions were measured per cell type. (C) AJ lengths, measured in randomly chosen optical fields of confluent cell cultures were assigned to categories as indicated, and the mean±s.e.m. number of corresponding AJ categories per 100 µm cell perimeter was determined for each cell type. The frequency distribution of AJ lengths per 100 µm (>250 junctions per cell type) is shown. **P<0.01, ***P<0.001, ^†^*P*<0.0001 (two-sample Kolmogorov–Smirnov test in B; Student's *t*-test in C).

To demonstrate that the observed alterations of cell–cell junctions were directly connected to plectin deficiency, we assessed the phenotype rescue potential of plectin isoforms found to be intrinsic to endothelial cells, specifically P1a and P1 (see Fig. S2). Indeed, the transient expression of GFP fusion proteins of either full-length P1a or P1, but not of GFP alone (control), led to a restoration of cell–cell junction continuity in transfected P0 cells (Fig. 3A). A statistical evaluation of AJ length distribution at transfected–untransfected versus untransfected–untransfected cell borders in subconfluent cell layers (∼48 h adhesion) showed that the forced expression of both, P1a and (to a lesser degree) P1, but not that of GFP alone, improved the continuity of AJs significantly, shifting the distribution of spike-like structures towards shorter lengths (Fig. 3B). Similarly, in confluent cell cultures, the frequency distribution of spike length categories per 100 µm of neighbouring transfected versus untransfected cells revealed a strong rescue potential for P1a, a lesser one for P1, but none for GFP alone, manifesting as an increase in the proportion of short spikes (<1 µm) and a decrease in that of longer ones (>2 µm) (Fig. 3C).

### Disrupted barrier function of P0 endothelial cells is rooted in their increased contractility and disorganization of vimentin filaments

**Fig. 4.**
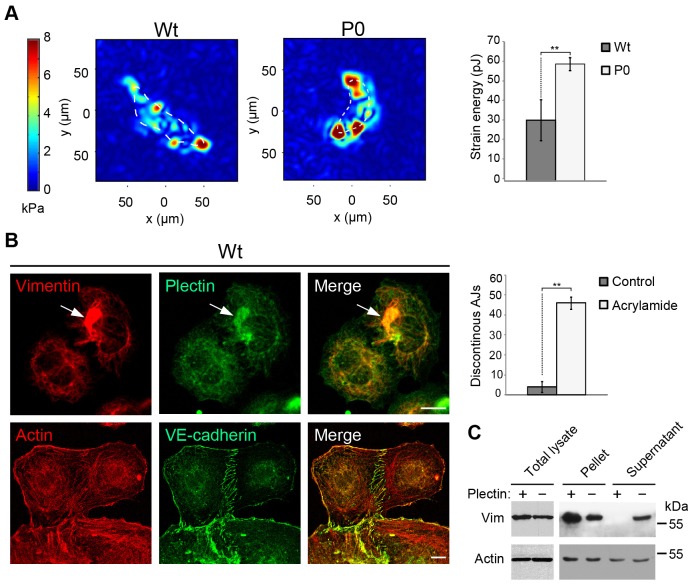
**Traction measurements and cytoskeletal alterations in P0 cells.** (A) Representative traction maps of Wt and P0 cells are shown in the left panels. Traction maps were computed from the displacement field of fluorescent beads embedded in collagen-coated polyacrylamide gels (before and after treatment with cytochalasin D), using FTTC. Colours in panels show the magnitude of the tractions in kPa (see colour bar). The bar graph shows the calculated strain energy from traction measurements. 57 Wt and 107 P0 cells were analysed from three independent experiments. **P<0.01 (Student's *t*-test). (B) Wt cells were seeded onto plates for 1 h in the presence of 6 mM acrylamide and processed for double immunofluorescence microscopy using antibodies to vimentin and plectin (upper row), or actin and VE-cadherin (lower row). The bar graph shows the quantitative evaluation (200 junctions per treatment, two independent experiments) of the number of cell–cell junctions exhibiting non-linear (‘zig-zag’) morphology along with actin stress fibres extending to the junctions (discontinuous AJs). Arrow (upper row), collapsed vimentin filaments colocalizing with plectin. Scale bars: 10 µm. **P<0.01 (Student's *t*-test). (C) Cells were harvested and either lysed using SDS buffer (total lysate), or incubated with detergent-extraction buffer, followed by centrifugation to yield detergent-insoluble pellets and detergent-soluble supernatants. Samples were analysed by immunoblotting using antibodies to the indicated proteins. Vim, vimentin.

Discontinuous cell–cell junctions lead to gaps between cells, increasing the permeability of the cell monolayer. It has been shown that such a condition is caused primarily by increased actin stress fibre contraction. To measure the contractile forces exerted by Wt and P0 endothelial cells we used traction force microscopy. As expected on the grounds of their more abundant actin stress fibre system, P0 endothelial cells exerted twofold higher traction forces and thus higher cell contractility than their Wt counterparts (Fig. 4A).

Apart from actin, it has been reported that changes in network organization of vimentin intermediate filaments and chemical modifications affecting their solubility have an impact on endothelial cell layer permeability (Liu et al., 2014, 2010; Shasby et al., 2002). Along these lines, by using a detergent-based cell fractionation assay, we could show that vimentin has increased extractability from P0 compared to Wt cells, whereas no changes were observed for actin solubility (Fig. 4C). To further assess whether vimentin network perturbations other than those caused by plectin deficiency would lead to a similar phenotype of discontinuous AJs, vimentin filaments in P0 cells were partially disassembled by incubating cells with acrylamide (Liu et al., 2014). Such treatment caused the retraction and collapse of vimentin intermediate filaments together with plectin around the nucleus, indicative of network disassembly (Fig. 4B, arrow). Concomitantly, we observed an increase in actin stress fibre formation and a dramatic increase in the number of distorted (discontinuous) AJs (to ∼50% of total junctions counted) (Fig. 4B, lower row and graph). These data strongly suggest that the vimentin intermediate filament network has to be intact and properly anchored to enable balanced stress fibre and cortical actin network formation, and consequently uninterrupted (linear) AJ formation.

### Reduced resilience of plectin-deficient endothelial cells towards mechanical stress

**Fig. 5.**
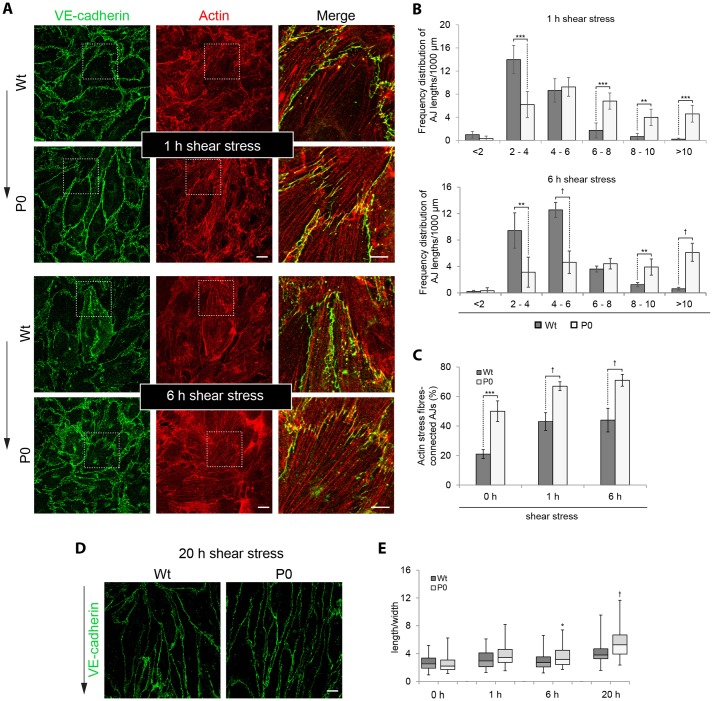
**Discontinuity of cell-cell junctions in P0 cells is potentiated by exposure to shear stress.** (A) Wt and P0 cells were exposed to flow shear stress (12 dyn/cm^2^) for 1 h or 6 h, and subjected to immunofluorescence microscopy using antibodies to VE-cadherin and actin. Merged images (third column) correspond to 3× magnified images of the boxed areas indicated in the first and second column. Arrow (outside of panel), flow direction. Scale bars: 10 µm (first and second column); 5 µm (third column). (B) AJ lengths were measured, assigned to different categories, and statistically evaluated as described in Fig. 2F. The frequency distribution of corresponding AJ lengths per 1000 µm total perimeters measured is shown. >1000 junctions per cell type and time point were evaluated. (C) Stress-fibre-connected AJs were evaluated as described in Fig. 2G. 0 h, 1 h and 6 h time points are shown. >1000 junctions were evaluated per cell type and time point. (D) Representative images of confluent Wt and P0 cells after 20 h shear stress exposure. Arrow (outside of panels), flow direction. Scale bar: 10 μm. (E) Ratio of longest length and widest diameter of cells (ImageJ, elongation factor) exposed to shear stress for the times indicated. Data are shown as box blot diagram (for details see Fig. 3B). >50 cells were measured per cell type and time point. **P<0.01, ***P<0.001, ^†^*P*<0.0001 (Student's *t*-test).

The finding of distorted AJs in plectin-deficient cells, in conjunction with the severe bleedings and haemorrhagic blisters observed in the skin of P0 but not of K5-Cre/cKO mice, suggested an increased fragility of the vasculature due to its reduced resistance towards mechanical stress in the absence of plectin. In order to compare the response of Wt and P0 cells to a mechanical stimulus, we used a shear stress assay where confluent cell layers were exposed to a controlled flow of medium for different time periods and afterwards subjected to immunolabelling for VE-cadherin and actin. As shown in Fig. 5A, flow exposure induced the formation of prominent and parallel-aligned actin stress fibres, especially in P0 cells. In addition, a marked shift towards more distorted AJs (characterized by longer VE-cadherin-positive spikes) was observed; the latter becoming most prominent in P0 cells at the 6-h time point (Fig. 5A,B). These observations are supportive of the notion that the massive build-up of stress fibres is potentiating the discontinuity of AJs at the posterior and anterior ends of the cell.

Concomitantly, the proportion of AJs with clearly evident actin stress fibre association increased with shear stress in both cell types, but was more pronounced in P0 cells (Fig. 5C). These drastic cytoskeletal rearrangements in P0 cells were accompanied by a more efficient orientation of cells in the flow direction, becoming particularly evident after 20 h of shear stress exposure (Fig. 5D). A statistical length-to-width ratio analysis revealed a significantly higher elongation of P0 compared to Wt cells after 6 h of treatment, further increasing up to 20 h (Fig. 5E).

**Fig. 6.**
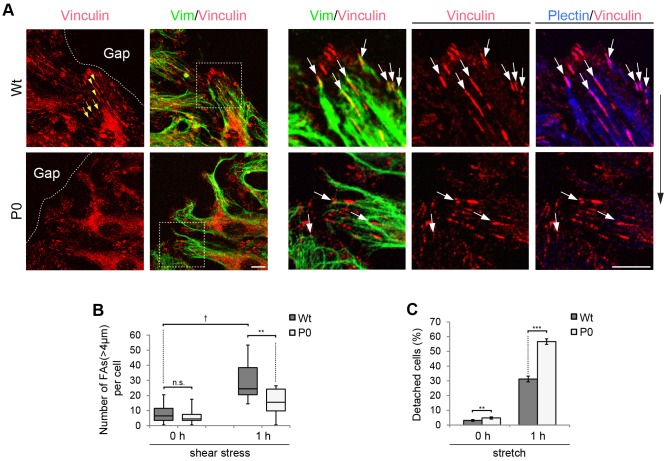
**Reduced mechanical resilience of P0 cells.** (A) Wt and P0 cells, exposed to flow shear stress (12 dyn/cm^2^) for 1 h, were subjected to immunofluorescence microscopy using antibodies to vinculin, vimentin (vim) and plectin (pseudocolours). Anti-vinculin staining and the merged image of anti-vimentin and anti-vinculin staining are shown in panels on the left (anti-plectin staining omitted). Dashed lines highlight cell monolayer-gap border. Arrowheads and arrows (in yellow), focal adhesions (FAs) proximal to and distal from the gap border, respectively. Boxed areas (inclusive staining for plectin) are shown at a higher magnification in panels on the right. Arrows (upper row), vimentin filaments orientated in the direction of the flow having their plectin-positive tips ending in vinculin-containing dash-like focal adhesions. Arrows (lower row), intermediate filaments overshooting focal adhesions or forming arch-like structures on top of them. Arrow outside of panels, direction of flow. Scale bars: 10 µm. (B) Quantitative evaluation of morphometric focal adhesion length analyses (ImageJ) in subconfluent cultures of immortalized cells prior to and after shear stress exposure for 1 h. >5000 focal adhesions in >20 cells were measured per cell type and time point; three independent experiments. Frequency distribution of focal adhesions with lengths of >4 µm per cell (all cells were roughly equal in size) is shown for no flow and 1 h of flow as a box plot diagram (for details see Fig. 3). (C) Wt and P0 endothelial cells were subjected to 30% cyclic stretching for 1 h at 0.25 Hz. The bar graph shows the mean±s.e.m. numbers of detached cells assessed by obtaining images before and after applying stretch; in control experiments no stretch was applied. Ten different areas from five different gels were assessed per cell type. **P<0.01, ***P<0.001, ^†^*P*<0.0001 (Student's *t*-test).

Cells withstand shear forces by increasing the size of focal adhesions, a process that has been shown to involve vimentin filaments attachment to these sites (Tsuruta and Jones, 2003). Accordingly, in regions of Wt cells that were exposed to elevated shear force, such as those next to sporadic gaps within the cell monolayer, we found that focal adhesions were much larger in size compared to focal adhesions located further away from the gap (Fig. 6A, first panel; yellow arrowheads versus arrows). In these regions vimentin filaments were found to be increasingly stretched with their tips terminating in vinculin-positive focal adhesions partially colocalizing with plectin (Fig. 6A, magnified panels, upper row, arrows). In the equivalent regions of the P0 cell layers, vimentin filaments appeared to overshoot focal adhesions, without indications of being anchored (Fig. 6A, magnified panels, lower row, arrows). A morphometric analysis of focal adhesion lengths after shear stress exposure revealed a substantial increase in the proportion of long (>4 µm) focal adhesions in Wt cells, and a comparatively moderate one in P0 cells (Fig. 6B). Thus, plectin-mediated vimentin intermediate filament network anchorage seems to be essential for the reinforcement of focal adhesions at shear-force-prone sites.

In a different type of assay, mechanical forces were imposed on cells by cyclic stretch, which results in cell detachment, allowing quantitative measurements. As expected, P0 cells showed significantly higher detachment rates than Wt cells (56% versus 29%), clearly indicating increased vulnerability (Fig. 6C). Thus both, shear stress and cyclic stretch assays, although differing in the type of forces applied on the cells, indicate a reduced mechanical resilience of P0 compared to Wt endothelial cells.

### Vascular phenotype assessment of plectin deficiency *in vivo*

**Fig. 7.**
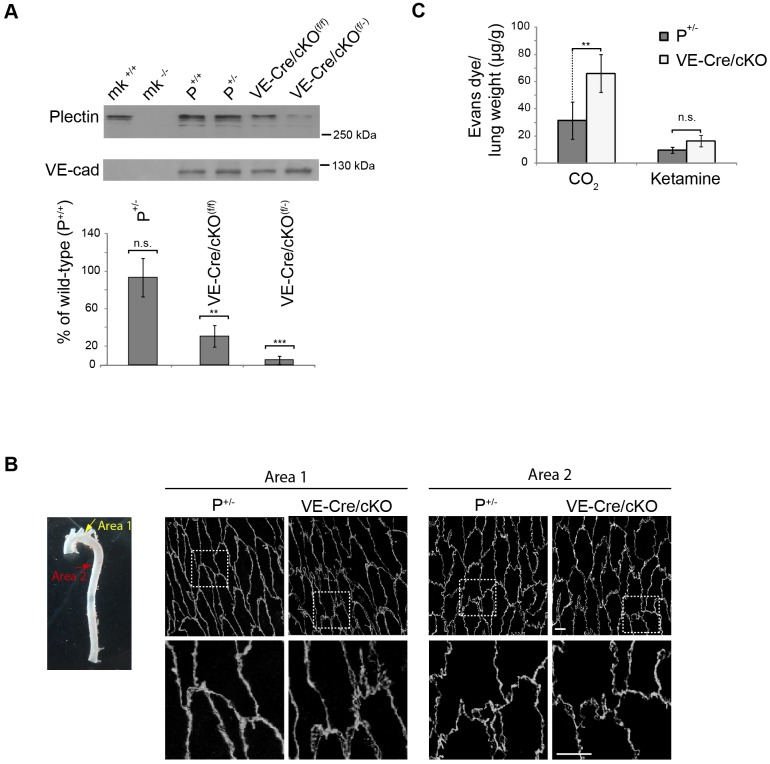
**Compromised barrier function of vasculature in VE-Cre/cKO mice.** (A) Immunoblotting of endothelial cell lysates prepared from lungs of mice carrying either two alleles of floxed plectin genes [P^+/+^ and VE-Cre/cKO^(f/f)^], or one null and one floxed allele of the plectin gene [P^+/−^ and VE-Cre/cKO^(f/−)^]. VE-Cre/cKO^(f/f)^ and VE-Cre/cKO^(f/−)^, Cre transgene expression. For immunoblotting, antibodies to plectin and VE-cad were used. Cell extracts from wild-type (mk^+/+^) and plectin-deficient (mk^−/−^) mouse keratinocyte cell lines (Osmanagic-Myers et al., 2006) served as VE-cadherin-negative controls. Blots were scanned and bands densitometrically quantified. The mean±s.e.m. (three independent experiments) values for plectin were plotted against those for VE-cadherin (used as an endothelial cell marker and loading control). Values are expressed as a percentage and are normalized to corresponding wild-type controls (P^+/+^). Note that (1) plectin levels in P^+/+^ and P^+/−^ mice are not significantly different, (2) plectin levels in VE-Cre/cKO^f/f^ and VE-Cre/cKO^f/−^ mice are reduced to ∼30% and ∼15%, respectively, and (3) VE-cadherin is not detectable in keratinocytes. (B) Representative *en face* VE-cadherin-specific immunolabelling in different areas of aortic endothelium from P^+/−^ and VE-Cre/cKO mice. Boxed areas are shown in lower panels at higher magnification. Scale bars: 10 µm. (C) The amount (µg) of Evans Blue dye extravasated from lungs of P^+/−^ and VE-Cre/cKO littermate pairs was calculated based on absorption values of standard series of the dye and normalized to the organ weight (g). Seven and three littermate pairs were killed with CO_2_, and ketamine and K^+^, respectively. **P<0.01; ***P<0.001; n.s., not significant (Student's *t*-test).

To study endothelium-associated phenotypes of plectin deficiency *in vivo*, we generated endothelium-restricted conditional knockout (cKO) mouse lines by breeding mice carrying either two floxed alleles (P^+/+^), or one null allele and one floxed allele of plectin (P^+/−^) (Ackerl et al., 2007), with transgenic mice expressing Cre recombinase driven by the endothelial-specific VE-cadherin promoter (Alva et al., 2006). Mice generated in this way (VE-Cre/cKO^f/f^ and VE-Cre/cKO^f/−^, respectively) were born at the expected Mendelian ratios, showed no overt phenotype and had a normal life span. To quantify plectin protein levels, lysates of lung endothelial cells, derived from either type of conditional knockout mice as well as from heterozygous plectin knockout (P^+/−^) and Wt (P^+/+^) littermate mice, were analysed by immunoblotting. The comparison relative to wild-type (P^+/+^) mice revealed a dramatic reduction of plectin protein levels in both types of cKO mice (to ∼30% and ∼15%, in VE-Cre/cKO^f/f^ and VE-Cre/cKO^f/−^, respectively), whereas no significant reduction was observed for heterozygous (P^+/−^) mice (Fig. 7A). The histological examination of blood vessel morphology and tissue architecture in heart, kidney, lung and liver revealed no evident differences between cKO and Wt littermates (data not shown). Because of its higher depletion in plectin, the VE-Cre/cKO^f/−^ mouse line (from now on referred to as VE-Cre/cKO) was chosen for a more detailed morphological analysis of cell–cell junctions in aortic endothelial cell sheets.

Consistent with previous reports (Chiu and Chien, 2011; Hakanpaa et al., 2015; Melchior and Frangos, 2010), *in situ* immunolabelling of P^+/−^ specimens for VE-cadherin revealed a mostly linear lining of cells in high-flow regions of the aortic arch outer curvature, whereas the patterns observed in regions exposed to lower flow forces in the descending aorta were more irregular (Fig. 7B, compare areas 1 and 2). Interestingly, in VE-Cre/cKO specimens, distortions and irregularities of cell–cell borders, particularly at the anterior and posterior edges of cells, were observed in both, high- and low-flow regions (areas 1 and 2 in Fig. 7B).

To test whether plectin deficiency affected the permeability of the vasculature in mutant mice, we performed the so called Evans Blue extravasation assay, which measures tissue uptake of an intravenously injected dye after the animal is killed (Martins-Green et al., 2008). When the mice were killed by CO_2_ inhalation, followed by organ removal and the amounts of dye taken up in the tissues were determined spectrometrically, all organs tested (heart, kidney, liver, lung and spleen) showed a tendency towards increased dye extravasation in VE-Cre/cKO compared to Wt littermates (data not shown). However, a significant increase was measured only in lung tissue (Fig. 7C, CO_2_). To eliminate the stress exerted on the lung tissue by CO_2_, we applied ketamine and K^+^ solution as an alternative method to kill the animals. In this case, dye uptake by the lung tissue was generally lower compared to the CO_2_ method (Fig. 7C, ketamine); nevertheless, a trend towards greater permeability of the mutant vasculature was still noticeable, although the difference no longer was significant. Overall, the outcome of these *in vivo* experiments was consistent with what one might have expected based on the cell–cell junction distortions and elevated permeability observed in cultured P0 cells.

## DISCUSSION

The endothelial cell cytoskeleton appears to be one of the key factors regulating endothelial barrier function. Particularly for the actin cytoskeleton, multiple studies have demonstrated the importance of its contractile function in exerting direct pulling forces on cell–cell junctions through actin stress fibres, leading to the formation of discontinuous cell–cell junctions and creation of intercellular gaps (Millan et al., 2010). By contrast, cortical actin structures are required for the formation of linear and continuous AJs, which are necessary for maintaining the barrier function of the endothelium (Dudek and Garcia, 2001). Thus, an increasing number of studies are focused on proteins and mechanisms that are able to maintain or shift the balance between these two types of actin structures, with the aim to regulate endothelial barrier properties and find a treatment for pathological conditions, such as inflammation, oedema and atherosclerosis.

Here, we report on the formerly unrecognized role of plectin, one of the major cytoskeletal linker proteins, in regulating the endothelium barrier function. Our study shows that plectin is crucial in keeping the actin filament system ‘in check’ maintaining the balance between continuous and discontinuous AJs. Using immunofluorescence, electron and traction force microscopy, we could demonstrate elevated actin stress fibre formation and increased cell contractility in plectin-deficient endothelial cells. Consequently, an increased number of discontinuous AJs (and TJs) was observed in these cells. We conclude that the intermittent nature of cell–cell junctions is the direct cause for the elevated permeability of endothelial P0 cell layers and the lung vasculature of VE-Cre plectin cKO mice.

What could be the mechanism behind the observed increase in actin stress fibres and distorted AJs, the perturbation of barrier properties and the lowering of the mechanical resilience of the endothelial cell system in the absence of plectin? Growing evidence shows that the vimentin filament system is indispensable for the endothelial stress response and barrier protection (Liu et al., 2014, 2010; Nieminen et al., 2006). Based on live imaging microscopy data showing an increase in directional displacements of vimentin filaments upon exposure to shear stress (Helmke et al., 2000), Conway and colleagues have used VE-cadherin- and platelet endothelial cell adhesion molecule (PECAM1)-FRET tension sensors to demonstrate that the vimentin filament networks bear a substantial amount of myosin-generated shear stress force to cell–cell junctions of endothelial cells (Conway et al., 2013). Furthermore, short-term flow-induced dilation of arteries and long-term flow-induced arterial remodelling are impaired in vimentin-knockout mice (Henrion et al., 1997; Schiffers et al., 2000).

The attachment of vimentin filaments to focal adhesions in endothelial cells has been shown to increase the size and adhesion strength of these structures (Bhattacharya et al., 2009; Tsuruta and Jones, 2003), similar to in fibroblasts (Burgstaller et al., 2010). Consequently, the absence of vimentin reduces the ability of endothelial cells to withstand high shear forces. In line with these observations, our study shows an increase in plectin-mediated attachment of vimentin filaments to focal adhesions and an increase in their length upon shear stress. P0 cells that lack such intermediate-filament–focal-adhesion connections exhibited reduced size enlargement of focal adhesions upon shear stress and detached more readily in stretching experiments compared to their wild-type counterparts. These data strongly suggest that the anchorage of vimentin filament networks at focal adhesions through plectin is a necessary prerequisite for endothelial cells to be able to withstand exposure to various kinds of mechanical stress. Plectin-mediated anchorage of vimentin intermediate filaments to focal adhesions has previously been shown to be necessary for vimentin networks to become organized in a cage-like structure encapsulating the nucleus (Wiche and Winter, 2011). This type of intermediate filament organization in turn acts as physical constraint for actomyosin gels, and its absence leads to a compensatory increase in actin stress fibres (Gregor et al., 2014). Showing that alterations of both the vimentin and the actin filament systems have wide-ranging consequences for the organization of cell–cell junctions, we provide here a novel mechanistic insight into endothelial cell barrier function. The observed increased intermittent character of AJs and TJs in the absence of plectin is a feature expected to lead to more ‘leaky’ junctions upon exposure to permeability-promoting stimuli such as thrombin, histamine, tumour necrosis factor and other growth factors (Dejana et al., 2009; Shasby et al., 2002).

**Fig. 8.**
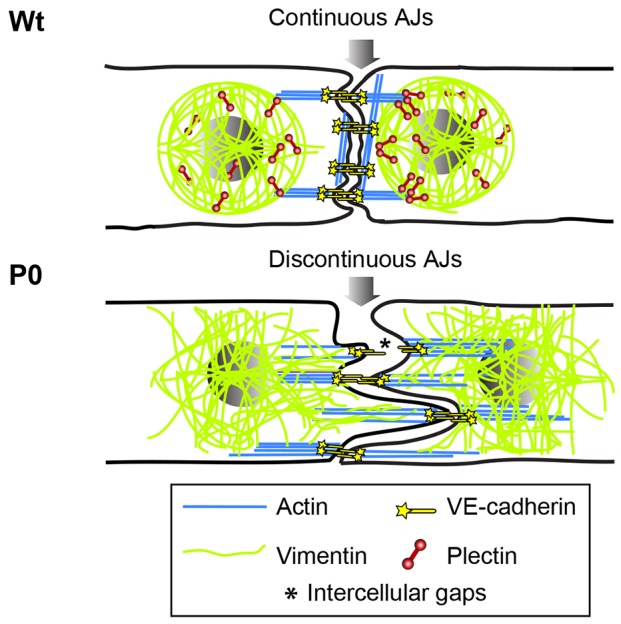
**Hypothetical model depicting the role of plectin as coordinator of cytoskeletal architecture and AJ organization in endothelial cells.** In monolayer-forming Wt endothelial cells (upper drawing), plectin is suggested to confine the vimentin network to central parts of the cells by internal crosslinking and anchoring to basal focal adhesions (not shown) underlying the nucleus. This presumably leads to a mechanically stable cage-like intermediate filament structure encapsulating and immobilizing the nucleus (see also Burgstaller et al., 2010; Wiche and Winter, 2011). With docking sites for actin (provided by plectin molecules) on its surface, the central intermediate filament network could serve as mechanical constraint counterbalancing actomyosin-generated forces and ensuring a uniform distribution of pulling forces acting through stress fibres on AJs along the cell borders. According to this model the mechanical constraint and the even distribution of contractile forces provided by intermediate filaments are essential prerequisites for the linear positioning of AJs between two neighbouring cells. Note that in regions with continuous AJs, cortical actin cables run parallel to the cell border, whereas at sites of AJ discontinuity they are oriented perpendicular to it. In the absence of plectin (P0, lower drawing), vimentin filaments (having lost their interlinkages and focal adhesion anchorage) extend to the very periphery, intruding in an unconstrained fashion the regions normally occupied by cortical actin networks. Concomitantly, vimentin-network-decoupled stress fibres are upregulated, leading to increased contractility and unequal distribution of pulling forces. As a result, AJs become discontinuous, favouring the formation of intercellular gaps between adhering cells.

The phenotypic changes observed in the absence of plectin might also be accompanied by alterations in signalling pathways, presumably due to the role of plectin as a scaffolding platform for different signalling molecules (Osmanagic-Myers et al., 2006). In our study, we started to tackle this issue by showing that the blocking of ROCK activity and the entailing reduction of actin stress fibres leads to a reduction of cell–cell junction distortions in P0 cells. This indicates that increased RhoA–ROCK activation is involved in the mechanism responsible for the phenotype, and future studies should address this issue by measuring RhoA activation in P0 cells and identifying its downstream effectors. Our results also show that the putative activation of ROCK is not accompanied by altered expression or localisation of the RhoA activator GEF Syx or the inhibitor RhoGAP190, suggesting that these upstream regulators are not directly responsible for the phenotype. Anyway, our findings that selective disruption of vimentin intermediate filaments shifts the cytoskeletal balance towards increased actin stress fibres and discontinuous AJs, and that of actin stress fibres (ROCK inhibition) towards continuous AJs, both confirm that a finely tuned interplay between actin and vimentin filament networks is required for proper AJ organization. Being closely associated with both the intermediate filament and the actin filament networks of endothelial cells, plectin thus emerges as a key factor in mediating the coordinated action of the cytoskeleton required for cell–cell junction homeostasis. This notion is supported by our observation that cell–cell junction distortions of P0 cells could be largely restored through forced expression of endothelial-cell-intrinsic isoforms of plectin. An operational model depicting the role of plectin as a coordinator of cytoskeletal architecture and AJ organization is shown and described in Fig. 8.

A number of our observations could be interpreted as implying that there is a crucial role of plectin for endothelial cells under conditions of increased mechanical strain. First, the reduced ability of P0 cells to enlarge their focal adhesions upon shear stress and the accompanying increase in cell–cell junction distortions might imply that there is a diminished cell resistance to shear forces. Second, pronounced differences between Wt and VE-Cre/cKO mice in cell–cell junction organization were observed in the outer curvature region of the aorta, where endothelial cells are exposed to increased shear stress (Van Doormaal et al., 2012). Third, the increased detachment rate of P0 compared to Wt cells when endothelial cell monolayers were subjected to cell stretch assays could be a reflection of the increased fragility of blood vessels leading to their rupture in blister-proximal regions. As the forces imposed on the cells under the conditions leading to these various phenotypes were different, it is likely that distinct cellular response mechanisms were involved. To elucidate these mechanisms in more detail and find possible common features should be a challenging task for future studies.

In summary, our study reveals new aspects of intermediate-filament-mediated regulation of endothelial cell contractility, one of the key targets in the treatment of cardiovascular diseases, including pulmonary hypertension, atherosclerosis and aortic stiffness (Fukumoto et al., 2005; Nohria et al., 2006; Noma et al., 2007). Our study links the intermediate-filament–plectin complex to an important barrier-protective function, particularly in stress-exposed endothelium, which is likely to be a prime target under endothelial injury conditions such as pulmonary oedema and acute respiratory distress syndrome. Treatments leading to an improvement of intermediate filament functionality might hence have promise in alleviating symptoms of EBS variants associated with respiratory distress.

## MATERIALS AND METHODS

### Generation of VE-Cre/cKO mice

Conditional plectin-knockout mice (VE-Cre/cKO) were generated by crossing mice bearing one null allele and one floxed allele of plectin (P^+/−^) (Ackerl et al., 2007) (back-crossed to the C57BL/6 background for 10 generations) with transgenic mice expressing Cre-recombinase driven by the endothelium-specific VE-promoter [The Jackson Laboratory, strain name: B6.Cg-Tg(Cdh5-cre)7Mlia/J, stock number: 00137] (Alva et al., 2006) (back-crossed to the C57BL/6 background for 12 generations). Animal studies were approved by the Federal Ministry for Science and Research (BMWF), Vienna, Austria.

### Isolation and immortalization of endothelial cells

Primary endothelial cell cultures were prepared from lungs of Wt and P0 newborn mice following a previously established protocol (Dong et al., 1997) modified such that the crude cell mixture isolated from lungs (collagenase I treatment) was first grown to confluence for 2–3 days prior to sorting with magnetic beads.

Polyoma middle-sized T-antigen (PmT) infection was used to specifically immortalize endothelial cells (Williams et al., 1988). A PmT-producing safe packaging fibroblast cell line GP+E-86 (Markowitz et al., 1988), stably transfected using a replication-defective neomycin selectable retrovirus, was kindly provided by Erwin Wagner (NCIO, Madrid, Spain). Key experiments performed with immortalized cells (cell junction morphology, permeability and shear stress assays) were confirmed using primary cell cultures without observing differences. All cases where exclusively primary cell cultures were used for experiments are explicitly specified; immortalized cell lines are referred to as Wt and P0 cells.

### Immunofluorescence microscopy

Cells grown overnight (or for 1 h) on gelatin (1% w/v) and fibronectin (1 µg/ml) coated plates, were fixed with methanol and processed for immunofluorescence microscopy as described previously (Rezniczek et al., 1998). The following primary antibodies were used: anti-plectin antiserum (#46, 1:400; Andrä et al., 2003), goat anti-VE-cadherin antibodies (sc-6458, Santa Cruz Biotechnology, 1:30), affinity-purified goat anti-vimentin antibodies (kindly provided by Peter Traub, Max-Planck-Institute, Ladenburg, Germany; 1:800; Giese and Traub, 1986), mouse monoclonal antibodies (mAbs) to vinculin (V9131, Sigma-Aldrich, 1:400), chicken anti-GEF Syx antibodies (kindly provided by Arie Horowitz, Cleveland Clinic, USA; 1:50), anti-GAP190 antiserum (kindly provided by Sarah Parsons, University of Virginia; 1:50), rabbit anti-ZO-1 antibodies (Invitrogen, 1:50) and rabbit affinity-purified antibodies to actin (A2066, Sigma-Aldrich; 1:100). As secondary antibodies, we used donkey anti-mouse-IgG and donkey anti-rat-IgG conjugated to Rhodamine Red-X, donkey anti-rabbit-IgG conjugated to DyLight 649, donkey anti-rabbit-IgG conjugated to Alexa Fluor 488 and donkey anti-goat-IgG conjugated to Alexa Fluor 488 (all from Jackson ImmunoResearch Laboratories). Specimens were viewed in an LSM 510 and LSM 710 laser-scanning microscope (Zeiss).

### Electron microscopy

Cells grown on Aclar coverslips underwent microwave-accelerated fixation in 0.5% glutaraldehyde, 0.1 M sodium cacodylate, pH 7.3 and were processed for electron microscopy as previously described (Reipert et al., 2008).

### Permeability assay

Cells were seeded onto Matrigel-coated 3-µm pore size filters of transwell inserts (2×10^5^/well) in 24-well plates and grown for 48 h. 10 µg/ml dextran–FITC (3 kDa) was added to the lower abluminal side of the chamber and, at regular time intervals, 10 µl aliquots were taken from the upper (luminal) part of the chamber and diluted with 90 µl water per well in a 96-well plate. After removing aliquots at the last time point, the fluorescence intensity of the aliquots was measured using a 96-well plate fluorimeter reader with excitation at 485 nm and emission at 535 nm as previously described (Martins-Green et al., 2008).

### Shear stress

15×10^4^ cells were seeded per channel of gelatin (1% w/v) and fibronectin (1 µg/ml)-coated flow chambers (80601, Ibidi), and left to adhere for ∼24 h. A flow generating a shear stress of 12 dynes/cm^2^ (similar to that in human arteries) was used in all experiments (DePaola et al., 1999).

### Acrylamide and ROCK inhibitor treatment

Cells were seeded onto plates for 1 h in the absence or presence of 6 mM acrylamide, fixed with methanol as described above, and processed for double immunofluorescence microscopy. For statistical evaluation of discontinuous AJs (non-linear or zig-zag morphology and actin stress fibres association), individual paired cells adhering to each other were monitored. Treatment of confluent (24 h-adhered) cultures with 20 µM ROCK inhibitor (Y-27632, Cell Guidance Systems) was performed for 1 h.

### Cell transfection

3×10^5^ cells were seeded per 35-mm plastic dish 24 h prior to transfection with expression plasmids encoding GFP-tagged versions of full-length P1a (pGR245), P1 (pGR260) (Andrä et al., 2003) and control plasmid pEGFP-N2 (Clontech). Transfection was carried out using Lipofectamine LTX Reagent and PLUS Reagent (Invitrogen), following the company's protocol optimised for human umbilical vein endothelial cells.

### Cell lysis and detergent extraction

For direct lysis, confluent endothelial cell cultures, washed twice with phosphate-buffered saline (PBS), were overlaid with 50 mM Tris-HCl, pH 6.8, 100 mM DTT, 2% SDS, 0.1% Bromophenol Blue and 10% glycerol. Detergent extraction of soluble vimentin was performed as described previously (Rezniczek et al., 2015). Aliquots of total cell lysates or cell fractions were subjected to SDS-PAGE and, after immunoblotting using peroxidase-coupled secondary antibodies, protein bands were visualized by exposure to X-ray film (Osmanagic-Myers and Wiche, 2004). For immunoblotting, the following primary antibodies were used anti-vimentin antibody (1:10,000; Giese and Traub, 1986), mouse mAbs B-5-1-2 to α-tubulin (Sigma-Aldrich; 1:10,000), rat mAbs to VE-cadherin (555289, BD Pharmingen; 1:500), mouse mAbs to actin (A4700, Sigma-Aldrich; 1:1000), anti-plectin isoform P1, P1a, P1c, and P1f antisera (Abrahamsberg et al., 2005; Rezniczek et al., 1998) and anti-plectin antiserum (#9, 1:400; Andrä et al., 2003). Secondary antibodies were horseradish-peroxidase-conjugated goat anti-rabbit-IgG, goat anti-mouse-IgG, goat anti-rat-IgG and donkey anti-goat-IgG (all from Jackson ImmunoResearch Laboratories; 1:20,000).

### Cell stretching and 2D traction microscopy

3×10^4^ cells were seeded onto flexible polydimethysiloxan gels coated with gelatin (1% w/v) and fibronectin (1 µg/ml) and left to grow overnight. Subconfluent cultures were subjected to uniaxial cyclic stretching (30% stretch) at 0.13 Hz for 1 h with a resting period of 1 s between lengthening and shortening phases as previously described (Bonakdar et al., 2012). For 2D traction microscopy, 10^5^ cells were seeded on acrylamide-bisacrylamide gels with embedded 0.5 µm green fluorescent beads. The forces that cells exert on their surroundings were measured by observing the displacements of fluorescent beads (Mierke et al., 2010). Direct computation of tractions was achieved by performing a Fourier decomposition of the displacements (Bonakdar et al., 2015).

### Evans Blue extravasation assay

Quantification of vascular leakage using the Evans Blue dye was performed as previously described (Matsuoka-Sakata et al., 2006), except for the following minor modifications: (1) the dye was allowed to circulate in live animals for 20 min and (2) animals were killed either using CO_2_ inhalation, or using intravenous injection of KCl (100 mg/kg) prior to being sedated by intraperitoneal injection of ketamine (100 mg/kg).

### Preparation of aortic tissue

Animals were killed by isoflurane inhalation, the chest cavity was cut open, and the aorta was exposed and washed free of blood by perfusion *in situ* with 2 mM EDTA in PBS, with the right atrium open to allow free flow. Dissected and longitudinally-slit aortas were pinned flat (with the intima surface up) on a silicone cell culture dish, fixed for 30 min in 4% paraformaldehyde and processed for immunofluorescence microscopy as described above.

### Statistical analysis

Data are presented as mean±s.e.m. Statistical analysis was carried out on data from independent experiments. For all experiments, *P* values were calculated using an unpaired two-tailed Student's *t*-test, except for Fig. 3B, where a two-sample Kolmogorov–Smirnov test was used.

## References

[JCS172056C1] Abrahamsberg C., Fuchs P., Osmanagic-Myers S., Fischer I., Propst F., Elbe-Bürger A. and Wiche G. (2005). Targeted ablation of plectin isoform 1 uncovers role of cytolinker proteins in leukocyte recruitment. *Proc. Natl. Acad. Sci. USA* 102, 18449-18454. 10.1073/pnas.0505380102 16344482 PMC1317913

[JCS172056C2] Ackerl R., Walko G., Fuchs P., Fischer I., Schmuth M. and Wiche G. (2007). Conditional targeting of plectin in prenatal and adult mouse stratified epithelia causes keratinocyte fragility and lesional epidermal barrier defects. *J. Cell Sci.* 120, 2435-2443. 10.1242/jcs.004481 17606998

[JCS172056C3] Alva J. A., Zovein A. C., Monvoisin A., Murphy T., Salazar A., Harvey N. L., Carmeliet P. and Iruela-Arispe M. L. (2006). VE-Cadherin-Cre-recombinase transgenic mouse: a tool for lineage analysis and gene deletion in endothelial cells. *Dev. Dyn.* 235, 759-767. 10.1002/dvdy.20643 16450386

[JCS172056C4] Andrä K., Lassmann H., Bittner R., Shorny S., Fässler R., Propst F. and Wiche G. (1997). Targeted inactivation of plectin reveals essential function in maintaining the integrity of skin, muscle, and heart cytoarchitecture. *Genes Dev.* 11, 3143-3156. 10.1101/gad.11.23.3143 9389647 PMC316746

[JCS172056C5] Andrä K., Kornacker I., Jörgl A., Zörer M., Spazierer D., Fuchs P., Fischer I. and Wiche G. (2003). Plectin-isoform-specific rescue of hemidesmosomal defects in plectin (-/-) keratinocytes. *J. Invest. Dermatol.* 120, 189-197. 10.1046/j.1523-1747.2003.12027.x 12542521

[JCS172056C6] Babic I., Karaman-Ilic M., Pustisek N., Susic S., Skaric I., Kljenak A. and Cikojevic D. (2010). Respiratory tract involvement in a child with epidermolysis bullosa simplex with plectin deficiency: a case report. *Int. J. Pediatr. Otorhinolaryngol.* 74, 302-305. 10.1016/j.ijporl.2009.10.002 20044146

[JCS172056C7] Bauer J. W., Rouan F., Kofler B., Rezniczek G. A., Kornacker I., Muss W., Hametner R., Klausegger A., Huber A., Pohla-Gubo G. et al. (2001). A compound heterozygous one amino-acid insertion/nonsense mutation in the plectin gene causes epidermolysis bullosa simplex with plectin deficiency. *Am. J. Pathol.* 158, 617-625. 10.1016/S0002-9440(10)64003-5 11159198 PMC1850321

[JCS172056C8] Bhattacharya R., Gonzalez A. M., DeBiase P. J., Trejo H. E., Goldman R. D., Flitney F. W. and Jones J. C. R. (2009). Recruitment of vimentin to the cell surface by beta3 integrin and plectin mediates adhesion strength. *J. Cell Sci.* 122, 1390-1400. 10.1242/jcs.043042 19366731 PMC2721003

[JCS172056C9] Bonakdar N., Luczak J., Lautscham L., Czonstke M., Koch T. M., Mainka A., Jungbauer T., Goldmann W. H., Schröder R. and Fabry B. (2012). Biomechanical characterization of a desminopathy in primary human myoblasts. *Biochem. Biophys. Res. Commun.* 419, 703-707. 10.1016/j.bbrc.2012.02.083 22386993

[JCS172056C10] Bonakdar N., Schilling A., Sporrer M., Lennert P., Mainka A., Winter L., Walko G., Wiche G., Fabry B. and Goldmann W. H. (2015). Determining the mechanical properties of plectin in mouse myoblasts and keratinocytes. *Exp. Cell Res.* 331, 331-337. 10.1016/j.yexcr.2014.10.001 25447312 PMC4325136

[JCS172056C11] Burgstaller G., Gregor M., Winter L. and Wiche G. (2010). Keeping the vimentin network under control: cell-matrix adhesion-associated plectin 1f affects cell shape and polarity of fibroblasts. *Mol. Biol. Cell* 21, 3362-3375. 10.1091/mbc.E10-02-0094 20702585 PMC2947472

[JCS172056C12] Castañón M. J., Walko G., Winter L. and Wiche G. (2013). Plectin-intermediate filament partnership in skin, skeletal muscle, and peripheral nerve. *Histochem. Cell Biol.* 140, 33-53. 10.1007/s00418-013-1102-0 23748243 PMC3695321

[JCS172056C13] Chiu J.-J. and Chien S. (2011). Effects of disturbed flow on vascular endothelium: pathophysiological basis and clinical perspectives. *Physiol. Rev.* 91, 327-387. 10.1152/physrev.00047.2009 21248169 PMC3844671

[JCS172056C14] Conway D. E., Breckenridge M. T., Hinde E., Gratton E., Chen C. S. and Schwartz M. A. (2013). Fluid shear stress on endothelial cells modulates mechanical tension across VE-cadherin and PECAM-1. *Curr. Biol.* 23, 1024-1030. 10.1016/j.cub.2013.04.049 23684974 PMC3676707

[JCS172056C15] Dejana E., Tournier-Lasserve E. and Weinstein B. M. (2009). The control of vascular integrity by endothelial cell junctions: molecular basis and pathological implications. *Dev. Cell* 16, 209-221. 10.1016/j.devcel.2009.01.004 19217423

[JCS172056C16] DePaola N., Davies P. F., Pritchard W. F. Jr., Florez L., Harbeck N. and Polacek D. C. (1999). Spatial and temporal regulation of gap junction connexin43 in vascular endothelial cells exposed to controlled disturbed flows in vitro. *Proc. Natl. Acad. Sci. USA* 96, 3154-3159. 10.1073/pnas.96.6.3154 10077653 PMC15911

[JCS172056C17] Dong Q. G., Bernasconi S., Lostaglio S., De Calmanovici R. W., Martin-Padura I., Breviario F., Garlanda C., Ramponi S., Mantovani A. and Vecchi A. (1997). A general strategy for isolation of endothelial cells from murine tissues: characterization of two endothelial cell lines from the murine lung and subcutaneous sponge implants. *Arterioscler. Thromb. Vasc. Biol.* 17, 1599-1604. 10.1161/01.ATV.17.8.1599 9301641

[JCS172056C18] Dudek S. M. and Garcia J. G. (2001). Cytoskeletal regulation of pulmonary vascular permeability. *J. Appl. Physiol.* 91, 1487-1500. 11568129 10.1152/jappl.2001.91.4.1487

[JCS172056C19] Fukumoto Y., Matoba T., Ito A., Tanaka H., Kishi T., Hayashidani S., Abe K., Takeshita A. and Shimokawa H. (2005). Acute vasodilator effects of a Rho-kinase inhibitor, fasudil, in patients with severe pulmonary hypertension. *Heart* 91, 391-392. 10.1136/hrt.2003.029470 15710736 PMC1768747

[JCS172056C20] Giese G. and Traub P. (1986). Induction of vimentin synthesis in mouse myeloma cells MPC-11 by 12-0-tetradecanoylphorbol-13-acetate. *Eur. J. Cell Biol.* 40, 266-274. 3519221

[JCS172056C21] Gregor M., Osmanagic-Myers S., Burgstaller G., Wolfram M., Fischer I., Walko G., Resch G. P., Jörgl A., Herrmann H. and Wiche G. (2014). Mechanosensing through focal adhesion-anchored intermediate filaments. *FASEB J.* 28, 715-729. 10.1096/fj.13-231829 24347609

[JCS172056C22] Hakanpaa L., Sipila T., Leppanen V.-M., Gautam P., Nurmi H., Jacquemet G., Eklund L., Ivaska J., Alitalo K. and Saharinen P. (2015). Endothelial destabilization by angiopoietin-2 via integrin beta1 activation. *Nat. Commun.* 6, 5962 10.1038/ncomms6962 25635707 PMC4316742

[JCS172056C23] Helmke B. P., Goldman R. D. and Davies P. F. (2000). Rapid displacement of vimentin intermediate filaments in living endothelial cells exposed to flow. *Circ. Res.* 86, 745-752. 10.1161/01.RES.86.7.745 10764407

[JCS172056C24] Henrion D., Terzi F., Matrougui K., Duriez M., Boulanger C. M., Colucci-Guyon E., Babinet C., Briand P., Friedlander G., Poitevin P. et al. (1997). Impaired flow-induced dilation in mesenteric resistance arteries from mice lacking vimentin. *J. Clin. Invest.* 100, 2909-2914. 10.1172/JCI119840 9389758 PMC508498

[JCS172056C25] Liu T., Guevara O. E., Warburton R. R., Hill N. S., Gaestel M. and Kayyali U. S. (2010). Regulation of vimentin intermediate filaments in endothelial cells by hypoxia. *Am. J. Physiol. Cell Physiol.* 299, C363-C373. 10.1152/ajpcell.00057.2010 20427712 PMC2928624

[JCS172056C26] Liu T., Ghamloush M. M., Aldawood A., Warburton R., Toksoz D., Hill N. S., Tang D. D. and Kayyali U. S. (2014). Modulating endothelial barrier function by targeting vimentin phosphorylation. *J. Cell Physiol.* 229, 1484-1493. 10.1002/jcp.24590 24648251

[JCS172056C27] Maniatis N. A. and Orfanos S. E. (2008). The endothelium in acute lung injury/acute respiratory distress syndrome. *Curr. Opin. Crit. Care* 14, 22-30. 10.1097/MCC.0b013e3282f269b9 18195622

[JCS172056C28] Markowitz D., Goff S. and Bank A. (1988). A safe packaging line for gene transfer: separating viral genes on two different plasmids. *J. Virol.* 62, 1120-1124. 2831375 10.1128/jvi.62.4.1120-1124.1988 PMC253118

[JCS172056C29] Martins-Green M., Petreaca M. and Yao M. (2008). An assay system for in vitro detection of permeability in human “endothelium”. *Methods Enzymol.* 443, 137-153. 10.1016/S0076-6879(08)02008-9 18772015

[JCS172056C130] Matsuoka-Sakata A., Tamura H., Asada H., Miwa I., Taketani T., Yamagata Y. and Sugino N. (2006). Changes in vascular leakage and expression of angiopoietins in the corpus luteum during pregnancy in rats. *Reproduction* 131, 351-360. 10.1530/rep.1.00947 16452728

[JCS172056C30] Melchior B. and Frangos J. A. (2010). Shear-induced endothelial cell-cell junction inclination. *Am. J. Physiol. Cell Physiol.* 299, C621-C629. 10.1152/ajpcell.00156.2010 20554908 PMC2944312

[JCS172056C31] Mellerio J. E., Smith F. J. D., McMillan J. R., McLean W. H. I., McGrath J. A., Morrison G. A. J., Tierney P., Albert D. M., Wiche G., Leigh I. M. et al. (1997). Recessive epidermolysis bullosa simplex associated with plectin mutations: infantile respiratory complications in two unrelated cases. *Br. J. Dermatol.* 137, 898-906. 10.1111/j.1365-2133.1997.tb01549.x 9470905

[JCS172056C32] Mierke C. T., Kollmannsberger P., Zitterbart D. P., Diez G., Koch T. M., Marg S., Ziegler W. H., Goldmann W. H. and Fabry B. (2010). Vinculin facilitates cell invasion into three-dimensional collagen matrices. *J. Biol. Chem.* 285, 13121-13130. 10.1074/jbc.M109.087171 20181946 PMC2857131

[JCS172056C33] Millan J., Cain R. J., Reglero-Real N., Bigarella C., Marcos-Ramiro B., Fernandez-Martin L., Correas I. and Ridley A. J. (2010). Adherens junctions connect stress fibres between adjacent endothelial cells. *BMC Biol.* 8, 11 10.1186/1741-7007-8-11 20122254 PMC2845098

[JCS172056C34] Ngok S. P., Geyer R., Liu M., Kourtidis A., Agrawal S., Wu C., Seerapu H. R., Lewis-Tuffin L. J., Moodie K. L., Huveldt D. et al. (2012). VEGF and Angiopoietin-1 exert opposing effects on cell junctions by regulating the Rho GEF Syx. *J. Cell Biol.* 199, 1103-1115. 10.1083/jcb.201207009 23253477 PMC3529520

[JCS172056C35] Nieminen M., Henttinen T., Merinen M., Marttila-Ichihara F., Eriksson J. E. and Jalkanen S. (2006). Vimentin function in lymphocyte adhesion and transcellular migration. *Nat. Cell Biol.* 8, 156-162. 10.1038/ncb1355 16429129

[JCS172056C36] Nohria A., Grunert M. E., Rikitake Y., Noma K., Prsic A., Ganz P., Liao J. K. and Creager M. A. (2006). Rho kinase inhibition improves endothelial function in human subjects with coronary artery disease. *Circ. Res.* 99, 1426-1432. 10.1161/01.RES.0000251668.39526.c7 17095725 PMC2666070

[JCS172056C37] Noma K., Goto C., Nishioka K., Jitsuiki D., Umemura T., Ueda K., Kimura M., Nakagawa K., Oshima T., Chayama K. et al. (2007). Roles of rho-associated kinase and oxidative stress in the pathogenesis of aortic stiffness. *J. Am. Coll. Cardiol.* 49, 698-705. 10.1016/j.jacc.2006.06.082 17291936 PMC2615568

[JCS172056C38] Osmanagic-Myers S. and Wiche G. (2004). Plectin-RACK1 (receptor for activated C kinase 1) scaffolding: a novel mechanism to regulate protein kinase C activity. *J. Biol. Chem.* 279, 18701-18710. 10.1074/jbc.M312382200 14966116

[JCS172056C39] Osmanagic-Myers S., Gregor M., Walko G., Burgstaller G., Reipert S. and Wiche G. (2006). Plectin-controlled keratin cytoarchitecture affects MAP kinases involved in cellular stress response and migration. *J. Cell Biol.* 174, 557-568. 10.1083/jcb.200605172 16908671 PMC2064261

[JCS172056C40] Reipert S., Kotisch H., Wysoudil B. and Wiche G. (2008). Rapid microwave fixation of cell monolayers preserves microtubule-associated cell structures. *J. Histochem. Cytochem.* 56, 697-709. 10.1369/jhc.7A7370.2008 18413652 PMC2430164

[JCS172056C41] Rezniczek G. A., de Pereda J. M., Reipert S. and Wiche G. (1998). Linking integrin alpha6beta4-based cell adhesion to the intermediate filament cytoskeleton: direct interaction between the beta4 subunit and plectin at multiple molecular sites. *J. Cell Biol.* 141, 209-225. 10.1083/jcb.141.1.209 9531560 PMC2132717

[JCS172056C42] Rezniczek G. A., Winter L., Walko G. and Wiche G. (2015). Functional and genetic analysis of plectin in skin and muscle. *Methods Enzymol.* [EPub] 10.1016/bs.mie.2015.05.003 26778562

[JCS172056C43] Schiffers P. M. H., Henrion D., Boulanger C. M., Colucci-Guyon E., Langa-Vuves F., van Essen H., Fazzi G. E., Levy B. I. and De Mey J. G. R. (2000). Altered flow-induced arterial remodeling in vimentin-deficient mice. *Arterioscler. Thromb. Vasc. Biol.* 20, 611-616. 10.1161/01.ATV.20.3.611 10712381

[JCS172056C44] Schröder R., Kunz W. S., Rouan F., Pfendner E., Tolksdorf K., Kappes-Horn K., Altenschmidt-Mehring M., Knoblich R., van der Ven P. F., Reimann J. et al. (2002). Disorganization of the desmin cytoskeleton and mitochondrial dysfunction in plectin-related epidermolysis bullosa simplex with muscular dystrophy. *J. Neuropathol. Exp. Neurol.* 61, 520-530. 12071635 10.1093/jnen/61.6.520

[JCS172056C45] Shasby D. M., Ries D. R., Shasby S. S. and Winter M. C. (2002). Histamine stimulates phosphorylation of adherens junction proteins and alters their link to vimentin. *Am. J. Physiol. Lung Cell. Mol. Physiol.* 282, L1330-L1338. 10.1152/ajplung.00329.2001 12003790

[JCS172056C46] Tsuruta D. and Jones J. C. R. (2003). The vimentin cytoskeleton regulates focal contact size and adhesion of endothelial cells subjected to shear stress. *J. Cell Sci.* 116, 4977-4984. 10.1242/jcs.00823 14625391

[JCS172056C47] Uehara K. and Uehara A. (2010). Vimentin intermediate filaments: the central base in sinus endothelial cells of the rat spleen. *Anat. Rec.* 293, 2034-2043. 10.1002/ar.21210 21089144

[JCS172056C48] Van Doormaal M. A., Kazakidi A., Wylezinska M., Hunt A., Tremoleda J. L., Protti A., Bohraus Y., Gsell W., Weinberg P. D. and Ethier C. R. (2012). Haemodynamics in the mouse aortic arch computed from MRI-derived velocities at the aortic root. *J. R. Soc. Interface* 9, 2834-2844. 10.1098/rsif.2012.0295 22764131 PMC3479906

[JCS172056C49] Wiche G. and Winter L. (2011). Plectin isoforms as organizers of intermediate filament cytoarchitecture. *Bioarchitecture* 1, 14-20. 10.4161/bioa.1.1.14630 21866256 PMC3158638

[JCS172056C50] Wiche G., Osmanagic-Myers S. and Castañón M. J. (2015). Networking and anchoring through plectin: a key to IF functionality and mechanotransduction. *Curr. Opin. Cell Biol.* 32, 21-29. 10.1016/j.ceb.2014.10.002 25460778

[JCS172056C51] Wildenberg G. A., Dohn M. R., Carnahan R. H., Davis M. A., Lobdell N. A., Settleman J. and Reynolds A. B. (2006). p120-catenin and p190RhoGAP regulate cell-cell adhesion by coordinating antagonism between Rac and Rho. *Cell* 127, 1027-1039. 10.1016/j.cell.2006.09.046 17129786

[JCS172056C52] Williams R. L., Courtneidge S. A. and Wagner E. F. (1988). Embryonic lethalities and endothelial tumors in chimeric mice expressing polyoma virus middle T oncogene. *Cell* 52, 121-131. 10.1016/0092-8674(88)90536-3 3345558

[JCS172056C53] Wright J. T., Fine J. D. and Johnson L. (1993). Hereditary epidermolysis bullosa: oral manifestations and dental management. *Pediatr. Dent.* 15, 242-248. 8247897

